# ET-1 as a Sex-Specific Mechanism Impacting Age-Related Changes in Vascular Function

**DOI:** 10.3389/fragi.2021.727416

**Published:** 2021-08-31

**Authors:** Andrew V. Kuczmarski, Laura M. Welti, Kerrie L. Moreau, Megan M. Wenner

**Affiliations:** ^1^ University of Delaware, Kinesiology and Applied Physiology, Newark, DE, United States; ^2^ University of Colorado, Anschutz Medical Campus, Aurora, CO, United States; ^3^ Denver Veterans Administrative Medical Center, Geriatric Research Education and Clinical Center, Aurora, CO, United States

**Keywords:** endothelin, aging, sex hormone, endothelial function, cardiovascular

## Abstract

Aging is a primary risk factor for cardiovascular disease (CVD), which is the leading cause of death in developed countries. Globally, the population of adults over the age of 60 is expected to double by the year 2050. CVD prevalence and mortality rates differ between men and women as they age in part due to sex-specific mechanisms impacting the biological processes of aging. Measures of vascular function offer key insights into cardiovascular health. Changes in vascular function precede changes in CVD prevalence rates in men and women and with aging. A key mechanism underlying these changes in vascular function is the endothelin (ET) system. Studies have demonstrated sex and sex hormone effects on endothelin-1 (ET-1), and its receptors ETA and ETB. However, with aging there is a dysregulation of this system resulting in an imbalance between vasodilation and vasoconstriction. Thus, ET-1 may play a role in the sex differences observed with vascular aging. While most research has been conducted in pre-clinical animal models, we describe more recent translational data in humans showing that the ET system is an important regulator of vascular dysfunction with aging and acts through sex-specific ET receptor mechanisms. In this review, we present translational evidence (cell, tissue, animal, and human) that the ET system is a key mechanism regulating sex-specific changes in vascular function with aging, along with therapeutic interventions to reduce ET-mediated vascular dysfunction associated with aging. More knowledge on the factors responsible for the sex differences with vascular aging allow for optimized therapeutic strategies to attenuate CVD risk in the expanding aging population.

## Introduction

Aging is a primary risk factor for cardiovascular disease (CVD), which is the leading cause of death in developed countries. Currently, 13% of the world population and 16% of the population in the United States is over the age of 65 (Ortman et al., 2014; Bank, 2019). Moreover, globally, the population of adults over the age of 60 is expected to double by the year 2050 (Ortman et al., 2014). The prevalence of CVD increases with age, but there are important sex differences in both prevalence and mortality (Virani et al., 2020). Young apparently healthy premenopausal women generally exhibit a favorable cardiovascular risk profile compared with age-matched men, largely attributed to the protective role of ovarian hormones (Iorga et al., 2017). However, the menopause transition is associated with an accelerated risk of CVD in women, matching or exceeding that of men (Dosi et al., 2014), and is thought to be due in part to changes in sex hormones. Understanding the mechanisms contributing to the sex differences in CVD with aging is important given differences in disease onset and mortality.

The endothelium is a monolayer of endothelial cells that creates a barrier between the blood and vascular smooth muscle cells (VSMC), forming the lumen of the blood vessel. To maintain homeostasis, the endothelium produces and releases both vasodilating and vasoconstricting factors, the most potent being nitric oxide (NO) and endothelin-1 (ET-1), respectively. Endothelial dysfunction, which precedes the development of cardiovascular pathologies such as atherosclerosis is a non-traditional risk factor for CVD (Vanhoutte et al., 2017). Endothelial dysfunction is characterized by a reduced vasodilatory capacity, primarily mediated by reduced NO bioavailability, a pro-inflammatory, and pro-thrombotic state of the vasculature (Bonetti et al., 2003). Measures of endothelial function are considered a biomarker for cardiovascular health, and functional changes in conduit and resistance vessels reflect changes in the coronary arteries (Muller-Delp, 2013; Schnabel et al., 2013; Huxley and Kemp, 2018), serving as a useful surrogate measure of coronary endothelial function.

**FIGURE 1:**
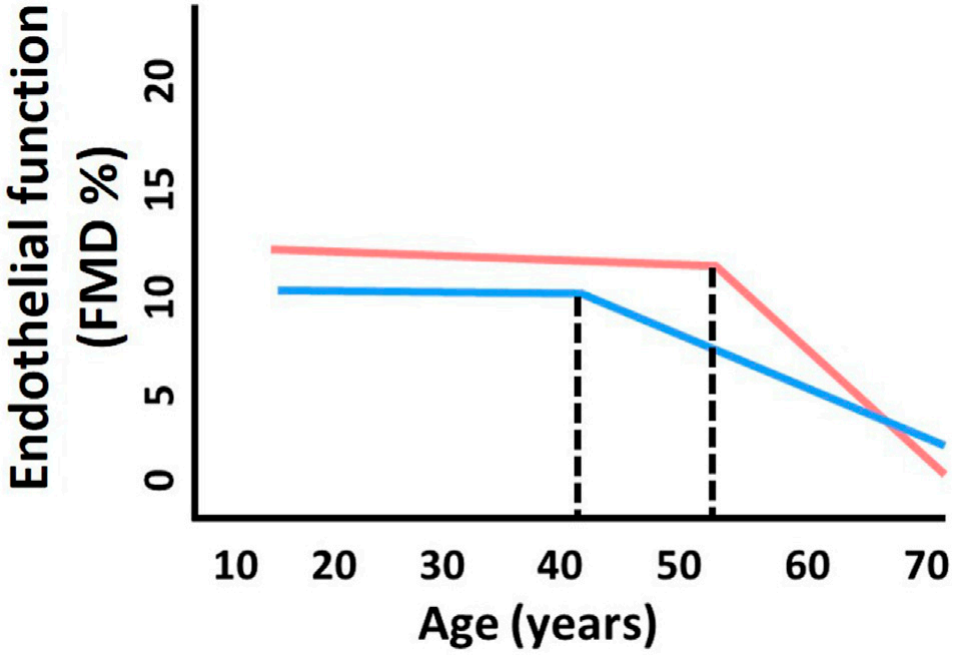
Sex differences in age-related declines in endothelial function. The decline in endothelial function (as measured by flow-mediated dilation, FMD) in men (blue) begins around the 4th decade of the life. This inflection point is shifted ∼10 years later in women (pink) to around the 5th decade of life, which is around the time of menopause. Thereafter, the rate of decline is greater in women compared to men with aging. Adapted from Celermajer et al. (1994).

Aging is associated with declines in endothelial function. Notably, there are sex differences in age-related declines in endothelial function. Young women tend to have greater endothelium-dependent dilation compared to men (Schnabel et al., 2013). In men, the decline in endothelial function, as measured by flow-mediated dilation (FMD), begins around the 4th decade of the life; however, this is delayed in women by ∼10 years occuring around the 5th decade of life (Celermajer et al., 1994), which is around the time of menopause (Figure 1). After menopause, the rate of decline is much greater in women compared to men as they age (Celermajer et al., 1994). These sex-specific declines in endothelial function with aging mirror the observed increase in CVD prevalence with aging. Importantly, endothelial function when used as a surrogate marker for cardiovascular health is a useful tool for studying sex differences in vascular aging.

The mechanisms underlying sex differences in the age-related decline in endothelial function remain under investigation, but accumulating evidence indicates that the ET system is a key sex-specific regulator of vascular dysfunction associated with aging. The ET system is active in many tissues such as neural, kidney, lung, and vasculature (Khimji and Rockey, 2010) and plays a role in cardiovascular physiology (Lerman et al., 1995) and numerous pathologies such as vascular dysfunction (Iglarz and Clozel, 2007), atherosclerosis (Ihling et al., 2001), hypertension (Cardillo et al., 1999), coronary artery disease (Kolettis et al., 2013; Best and Lerman, 2000) and heart failure (Ibrahim et al., 2018). Sex hormones can regulate the ET system at the cellular level leading to functional changes in the vasculature. Eloquent reviews have been published on sex differences in the ET system in hypertension, cardiovascular, and renal diseases (Montezano et al., 2008; Gohar and Pollock, 2017). Moreover, sex hormones can directly impact the ET system as previously reviewed (Mikkola et al., 1998; Tostes et al., 2003; Kittikulsuth et al., 2013). This review will focus on the ET system as a major contributor to sex-specific differences in vascular aging. We will review the role of ET-1 in vascular aging from a translational perspective, and update information on sex differences in the ET system with an emphasis on the therapeutic role of ET-1 in vascular adaptations to aerobic exercise.

Aging also increases the risk for CVD through other adverse effects on the arterial wall, including VSMC growth, apoptosis, vascular hypertrophy and enhanced matrix metalloproteinase activity, which can lead to arterial remodeling and stiffening (Folkow and Svanborg, 1993; McEniery et al., 2005; Cockcroft and Mancia, 2012). In contrast to age-associated endothelial dysfunction, there are disparities in the literature on whether there are sex differences in the age-related increase in arterial stiffness, with some studies reporting a similar progression in the age-related increase in arterial stiffness, a steeper progression in men after the age of 50, and greater age-related rates of aortic and carotid artery stiffening in women (DuPont et al., 2019). The disparities in the literature can be attributed to methodology, however, there is a lack of knowledge on how changes in sex hormones contribute to age-related arterial stiffening in both women and men. Although the focus of this review will be on endothelial function, we wish to refer the reader to these excellent reviews on arterial stiffness (Safar, 2018; DuPont et al., 2019).

## Regulation of Endothelin

The ET peptide is a potent vasoconstrictor primarily released from endothelial cells (Yanagisawa et al., 1988). There are three variants (ET-1, ET-2, ET3) of the vasoactive peptide with ET-1 being the most biologically active. ET-1 acts locally in a paracrine and autocrine manner due to its abluminal secretion and location of its receptors (Wagner et al., 1992; Davenport et al., 1990), and approximately 60% of circulating ET-1 is cleared in each pass by ETB receptors located on the lungs, kidney and liver, thus plasma concentrations are kept low (Frelin and Guedin, 1994; Dupuis et al., 1996; Johnström et al., 2005).

ET-1 synthesis is regulated through gene transcription. Gene transcription results in prepro-ET-1 mRNA which is cleaved by the protein convertase, furin, to yield Big ET-1 (Peto et al., 1996). Big ET-1 is a 38 amino acid peptide and is the precursor to the 21 amino acid long, biologically active ET-1 peptide (Russell et al., 1998). Big ET-1 can be cleaved by either of two endothelin converting enzymes, ECE-1 or ECE-2 (Turner and Murphy, 1996). ECE-1 has four splice variants and is present on both the plasma membrane and intracellular compartments of endothelial cells (Xu et al., 1994; Schweizer et al., 1997; Valdenaire et al., 1999). Additionally, there are mechanisms of ET-1 generation that are independent of ECE; ET-1_1-31_ is an inactive intermediate which is converted to active ET-1 neprilysin (Wypij et al., 1992; Maguire and Davenport, 2004). For a comprehensive review of endothelin synthesis, we refer the reader to the following publications (D’Orléans-Juste et al., 2003; Houde et al., 2016).

Following synthesis, the peptide and its precursor Big ET-1 are stored in intracellular vesicles such as Weibel-Palade bodies (Kavashima et al., 1999). A majority of the peptide is stored as Big ET-1 and is converted to the biologically active peptide during exocytosis. ET-1 has both a constitutive and regulated pathway for release (Seed et al., 2012). In the constitutive pathway, secretory vessels containing Big-ET1, ET-1, ECE-1, and ECE-2 are trafficked to the membrane for release towards the VSMC (Kuruppu et al., 2007). In the regulated pathway, ET-1 is stored in Weibel-Palade bodies of endothelial cells (Russell et al., 1998). Upon stimulation, trafficking to the membrane occurs and ET-1 is released abluminally providing an immediate increase in the local concentration of ET-1. This local surge in ET-1 following stimulation is important to its vasoactive properties in regulating vascular tone.

Although the endothelium is the primary source of ET-1, there are also non-endothelial sources of ET-1 that include VSMCs and immune cells such as monocytes, neutrophils, and mast cells. VSMCs release ET-1 at a rate that is 100× less than endothelial cells and do not utilize the same storage mechanisms of ET-1 as endothelial cells (Kanse et al., 1991). Despite the lower release of ET-1 from VSMC compared to endothelial cells, ET-1 expression can still be upregulated in VSMC in pathological conditions such as atherosclerosis (Haug et al., 1996). The cells can act through various feedback loops to influence ET-1 production, as demonstrated by the ability of VSMC to inhibit endothelial cell ET-1 production through a NO-mediated mechanism (Di Luozzo et al., 2000). Additionally, vascular cells can respond in unique ways to various ET-1 secretagogues. For example, endothelial cell and VSMC respond differently to ET-1 secretagogues, such as cortisol, which can increase VSMC ET-1 expression while not impacting endothelial cell ET-1 production (Kanse et al., 1991). Non-vascular cells such as immune cells express ET receptors and can release ET-1 to interact locally with the endothelium and VSMC (Iwasa et al., 1999; Elisa et al., 2015; Shinagawa et al., 2017). Specifically, immune cells interact with the endothelium and can increase local inflammation and lead to development of atherosclerotic plaques (Iwasa et al., 1999). Thus, the regulation of ET-1 is an amalgamation of factors including the stimuli, cell types stimulated, and individual cellular responses to secretagogues as well as local cell-to-cell feedback mechanisms.

ET-1 plays an essential role in regulating vascular tone through a balance of endothelial cell and VSMC receptor activation. In the vasculature, both the ETA and ETB receptors are expressed on VSMC and mediate vasoconstriction (Ivey et al., 2008; Nilsson et al., 2008). The vascular smooth muscle ETA receptor is the primary receptor driving the potent vasoconstrictor effects of ET-1 in healthy vasculature, which are long lasting. The signaling cascade involves phospholipase C (PLC), inositol triphosphate (IP3), and diacylglycerol (DAG) leading to an increase in intracellular calcium release (Avedanian et al., 2010). Other signaling pathways activated include the phospholipase D (PLD), phospholipase A2 (PLA2), and mitogen activated protein kinase (MAPK) contributing to the indirect effects of ET-1 such as cellular migration and proliferation (Khalil, 2011). ETA receptors also undergo posttranslational modification such as palmitoylation (Horstmeyer et al., 1996), however they do not undergo ligand-induced phosphorylation which contributes to their prolonged pressor effects (Cramer et al., 1997). The VSMC ETB receptor also leads to vasoconstriction acting through the PLC pathway (Wynne et al., 2009). The ETB receptors are secondary to the ETA receptor-mediated contraction of the VSMC, and expression of ETB receptors is low in non-pathological conditions. However, in pathological conditions ETB receptor expression is upregulated and can contribute to increased vascular tone (Donnelly, 2017).

The ETB receptors are also located on endothelial cells. Endothelial ETB receptors balance the vasoconstrictor effects of VSMC, promote vasodilation via enhancing NO and prostacyclin (PGI_2_), and act as a clearance receptor to help remove local ET-1 (Hirata et al., 1993). The ETB receptor has numerous sites for posttranslational modifications including phosphorylation, palmitoylation, and glycosylation which can alter its functional properties (Davenport et al., 2016). ETB receptors are deactivated via phosphorylation causing a reduction in activity within 5 minutes (Cramer et al., 1997) and are internalized and trafficked to an endosomal/lysosomal pathway for degradation (Bremnes et al., 2000). These properties of the ETB receptor enable it to act as a clearance receptor and attenuate the vasoconstrictor effects of ET-1 (Kelland et al., 2010). Additionally, the endothelial cell ETB receptor mediates vasodilation through activation of intracellular signaling pathways including PLC, phosphatidylinositol 3- kinase (PI3K), and second messengers IP3 and DAG leading to endothelial nitric oxide synthase (eNOS) phosphorylation and subsequent NO production (Shihoya et al., 2016). However, when there is increased oxidative stress, posttranslational modification of the ETB receptor can lead to attenuated NO production which can promote vascular pathologies (Maron et al., 2012). The impact of endothelin receptors on the vasculature depends on their level of expression and functional capacity, both of which can independently be impacted by aging, sex, and sex hormones.

## ET-1, Aging, and Sex

Aging, sex, and sex hormones are associated with changes in ET-1 synthesis, expression, release from endothelial cells, and circulating plasma levels, all which can contribute to vascular dysfunction (Tokunaga et al., 1992; Kumazaki et al., 1994; Donato et al., 2009). Moreover, ETA and ETB receptor expression and function differ between sexes, are modulated by changes in sex hormones, and impacted by aging (Van Guilder et al., 2007; Stauffer et al., 2010; Wenner et al., 2017; Sebzda et al., 2018; Stanhewicz et al., 2018). Directly measuring ET-1 and its receptors at the cell and tissue level can provide unique insights into the molecular mechanisms that are underlying changes in the ET system with aging. Increased ET-1 production and release with aging may be associated with increased receptor expression and activation, leading to a shift in vascular tone away from favoring vasodilation and towards a vasoconstrictor phenotype. This vasoconstrictor phenotype is pro-atherosclerotic and contributes to CVD onset and progression (Vanhoutte et al., 2009). Thus, the ET system may be used as a sex-specific biomarker of vascular health and serve as a molecular target for treatments to reduce age-related CVD.

### Plasma ET-1

Plasma levels of ET-1 increase with age and are elevated in middle-aged and older adults compared to young adults (Miyauchi et al., 1992). In women there is a stepwise increase in plasma ET-1 between young, middle, and older adults (Maeda et al., 2003). In men, there is also higher plasma ET-1 in older compared to younger adults (Donato et al., 2009). Plasma ET-1 levels have also been shown to be higher in men compared to women in both young and middle-aged adults (Miyauchi et al., 1992; Polderman et al., 1993). Although plasma ET-1 is higher in men, an 8-year observational study of Swedish adults (30–74 years) reported a strong association between plasma ET-1 and incident CVD in women, while observing no differences in baseline plasma ET-1 between men who did or did not develop incident CVD (Daka et al., 2015). Furthermore, a longitudinal observational cohort study showed plasma ET-1 was a predictor of 10-year all-cause mortality in a general population of healthy middle-aged and older adults (Yokoi et al., 2012). This finding was further supported in a study of polish centenarians in which probability of 1-year survival was associated with ET-1, suggesting a lower plasma ET-1 is a significant independent predictor for longer survival in centenarians (Szewieczek et al., 2015). Throughout aging those with cardiovascular risk factors, such as high blood pressure and atherosclerosis, appear to have elevated plasma ET-1 compared to their age-matched healthy controls (Lerman et al., 1991; Bossard et al., 2015) which could increase their risk of adverse outcomes (Zhang et al., 2017). Additionally, pathologies that are predictive of future CVD such as erectile dysfunction in men and preeclampsia in women, as well as smokers (Hirai et al., 2004), patients with insulin resistance (Piatti et al., 1996), or hyperlipoproteinemia (Haak et al., 1994) also demonstrate increased plasma ET-1, further supporting the importance of the ET system in CVD (El Melegy et al., 2005; George and Granger, 2011). This elevated plasma ET-1 in CVD is likely mediated by increased ET-1 production (synthesis and release) rather than a decrease in clearance (Parker and Thiessen, 2004).

The precursors (such as Big ET-1) and intermediates of ET-1 have also been measured in the plasma, and higher levels are associated with the presence of CVD pathology and severity (Leslie et al., 2004). The 31-amino acid intermediate of ET-1 (ET-1_1-31_) is produced through a chymase enzymatic reaction and is also biologically active although less potent than ET-1 produced through its traditional pathway (Leslie et al., 2004b; Maguire et al., 2001; Fecteau et al., 2005). Plasma levels of ET-1_1-31_ were higher in patients with atherosclerosis compared to healthy patients (Suzaki et al., 2003). ET-1_1-31_ is converted to active ET-1 by NEP, and inhibition of NEP can regulate the activity of ET-1_1-31_. A detailed review of ET-1_1-31_ and cardiovascular pathologies, which further describe the impact and significance of ET-1_1-31_ has previously been published (D’Orléans-Juste et al., 2008). Additionally, C-terminal pro-ET-1 (CT-proET-1) is crucial for ETA and ETB receptor activity, and can be found in the plasma and is elevated in cardiovascular pathologies and associated with organ damage in numerous physiological systems (Habib et al., 2010; Drion et al., 2012; Buendgens et al., 2017). Patients with high levels of CT-proET-1 also had higher mortality suggesting there could be a threshold effect or dose-response relation to disease severity and plasma CT-proET-1 (Buendgens et al., 2017). An additional benefit of measuring CT-proET-1 is the increased stability compared to ET-1 (Xu et al., 1994; Buendgens et al., 2017). Each peptide independently appears to be associated with CVD and collectively measuring plasma concentrations of ET-1, its variant ET-1_1-31_, and its precursors Big ET-1 and CT-proET-1 could provide a more comprehensive assessment of the ET system and its potential impact on cardiovascular health.

### Vascular ET-1 Expression and Sensitivity

Despite the benefits of measuring plasma ET-1, plasma concentrations of ET-1 may not always be reflective of tissue or cellular ET-1; local concentrations of ET-1 are tissue dependent and can be 100-fold higher than plasma concentrations (Battistini et al., 1993; Davenport et al., 2016). In animal models, aging is associated with increased expression of prepro-ET-1 mRNA, increased expression of ECE-1, greater stimulated release of ET-1, (Goel et al., 2010) and greater ET-1 expression in the coronary artery (Granado et al., 2014). Aging increases the number of secretagogues that can stimulate the ET system leading to increased activity. Numerous studies using either aging models in cell culture or using cells from older adults demonstrate an increased release of ET-1 in older compared to younger groups (Tokunaga et al., 1992; Kumazaki et al., 1994; Khatib et al., 2007; Wang et al., 2015). A greater concentration of ET-1 may contribute to the reduced sensitivity to ET-1, as noted by a reduced vasoconstrictor response to exogenous ET-1, although this may differ across vascular beds (Weinheimer et al., 1990; Homma et al., 1992; Donoso et al., 1994; Donato et al., 2005).

There is evidence in both animals and humans of reductions in ET-1 sensitivity with aging, which could suggest age-related increases in ET-1 expression and augmented endothelin-mediated vasoconstrictor tone. In humans, blunted vasoconstrictor responses to exogenous ET-1 have been reported in older compared to younger men (Van Guilder et al., 2007), suggesting increased vasoconstrictor tone attributable in part by ET-1 (Thijssen et al., 2007; Van Guilder et al., 2007; Westby et al., 2011). Moreover, in older men there is greater arterial and venous endothelial cellexpression of ET-1 compared to young men (Donato et al., 2009). Notably, there was an inverse relation between FMD and endothelial ET-1 expression, suggesting that the age-related decline in endothelial function in older men may be related to a greater expression of ET-1 and increased ET-1 signaling. Taken together, we speculate that the increased expression of ET-1 in endothelial cells (Donato et al., 2009) coupled with the blunted vasoconstrictor response to exogenous ET-1 in older men (Van Guilder et al., 2007) could suggest greater bioavailability of ET-1 in older adults. Importantly, comparable studies have yet to be published regarding the impact of aging on cellular ET-1 expression in women as well as if sensitivity to exogenous ET-1 is altered with aging in women. Sex differences in cellular secretion of ET-1 have been previously demonstrated. In a model examining male and female HUVECs from twins, males had higher levels of ET-1 secretion at rest. However, when stimulated with VEGF females had a significant increase in secreted ET-1, an effect that was not observed in the male cells, suggesting sex differences in the response to ET-1 secretagogues (Lorenz et al., 2019). Thus, there may be inherent biological differences in ET-1 release that lead to females secreting more ET-1 upon stimulation which may exacerbate endothelin-mediated dysfunction when ET-1 secretagogues increase with aging.

### ET-1 Interaction With Other Vasoactive Substances

ET-1 interacts with a number of other substances within the vasculature, such as NO, PGI_2_, and thromboxane. NO is a regulator of ET-1, and increased NO can downregulate ET-1 production (Bilsel et al., 2000; Tan et al., 2007). This finding is further supported through addition of L-NMMA, a NO inhibitor which led to increased ET-1 production (Akishita et al., 1998). Overexpression of ET-1 can modulate the production of vasodilators, while also exacerbating its pressor effects leading to increased vascular tone and reduced vasodilatory capacity. This can be observed in part through ET-1-mediated reduction in NO bioavailability due to impaired NO production and reduced expression of eNOS (Sud and Black, 2009). ET-1 also mediates superoxide production through activation of NADPH oxidase which can further inhibit vasodilator capacity (Sud and Black, 2009). With aging the increase in ET-1 is associated with a corresponding reduction in NO, both of which contribute to the age-associated decline in endothelial function. Moreover, prostanoid production shifts with aging to favor vasoconstriction (Seals et al., 2011; Bachschmid et al., 2013), and ET-1 upregulates cyclooxygenase-2 expression to favor inflammation and vasoconstriction (Lin et al., 2014). Sex hormones, ET-1 and aging are known to act on the vascular cyclooxygenase system and thus modulate vascular reactivity (Mikkola et al., 1998; Davidge 2001). Aging is also associated with an increased inflammation, characterized by increased proinflammatory cytokines including TNF-α, IL-6, and VCAM-1 as well as CRP and NF-kB (Donato et al., 2008; Donato et al., 2007). These inflammatory cytokines can stimulate ET-1 synthesis, further exacerbating the production of inflammatory cytokines and endothelial dysfunction. More comprehensive reviews on aging and NO can be found here (Seals et al., 2011; Cau et al., 2012; Bachschmid et al., 2013; Sverdlov et al., 2014).

## ETA and ETB Receptors

As previously mentioned, ETA and ETB receptors are located on VSMC, and the ETB receptor is also located on the endothelium. The ratio of receptors is important in mediating the functional responses to ET-1. There is a shift in ETA:ETB receptor expression with aging in male Sprague-Dawley rats which can lead to functional changes (Del Ry et al., 2008). In animal models of hypertension and hypercholesterolemia, the ratio of endothelial to smooth muscle ETB receptors is attenuated favoring a vasoconstrictor profile (Kakoki et al., 1999), and can contribute to ET-mediated vascular dysfunction. Importantly with aging there is an increased likelihood for developing hypertension and hypercholesterolemia, thus the changes in the ET system could be compounded by age as well as CVD pathologies.

In humans, sex and aging can both impact receptor density and function. The ETB receptor mediates vasoconstrictor tone in young men, whereas it mediates vasodilator tone in young women (Kellogg et al., 2001; Wenner et al., 2017). These sex differences are also apparent in older adults; Stauffer and colleagues demonstrated older men have greater ETA mediated vasoconstrictor tone compared to women (Stauffer et al., 2010). Interestingly, older postmenopausal women had greater increases in forearm blood flow to dual antagonism of the ETA and ETB receptor compared to ETA alone, suggesting ETB receptors play a larger role in mediating vascular function in older women while ETA receptors play a larger role in older men (Stauffer et al., 2010). Additionally, receptor density is different between older men and women such that older men have a greater density of ETA receptors as well as a higher ratio of ETA:ETB receptors in the saphenous vein compared to women (Ergul et al., 1998). A lack of ETB-mediated vasodilation and increased ETA-mediated vasoconstriction can reduce the vasodilatory capacity contributing to vascular dysfunction. The shift in receptor expression is compounded by changes in ET-1 expression that further exacerbates ET-mediated dysfunction. Collectively, these differences in receptor expression result in functional changes that contribute to age-related endothelial dysfunction.

### Women

**FIGURE 2:**
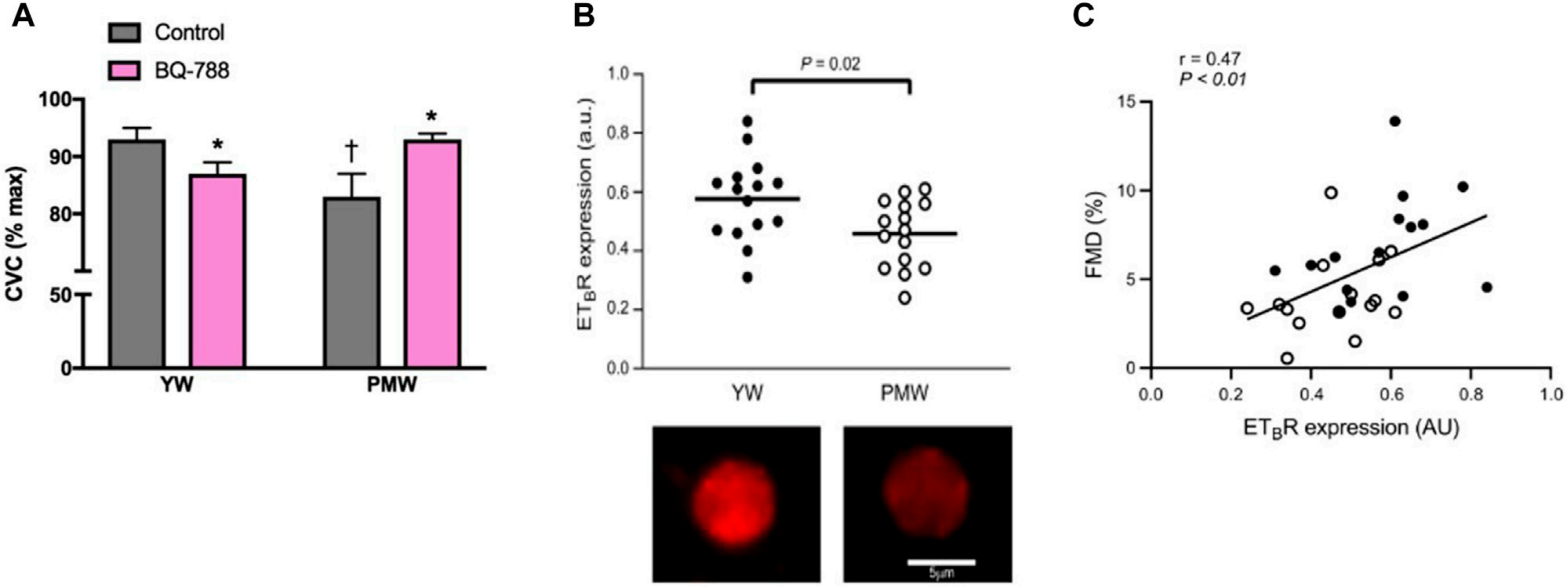
Loss of ETB-mediated vasodilation and decreased endothelial ETB receptor expression contribute to age-related declines in endothelial function in women. Cutaneous microvascular conductance (CVC; expressed as % maximal dilation) was measured in young women (YW) and postmenopausal women (PMW) during local heating with microdialysis perfusions of lactated Ringer solution (control; gray bars) or the ETB receptor antagonist BQ-788 (pink bars). ETB receptors mediate dilation in young women (YW), an effect that is lost in postmenopausal women (PMW) **(A)** Graph from Stanhewicz et al. (2018); data from Wenner et al. (2017). ETB receptor expression [in arbitrary units (a.u.)] in venous endothelial cells collected from healthy young women (YW) and postmenopausal women (PMW). **(B, top)** Representative images of a venous endothelial cell ETB receptor expression in a YW and PMW with a scale bar representing cell size **(B, bottom)** Data from Kuczmarski et al. (2020). Pearson correlation showing the significant relation between flow-mediated dilation (FMD, %) and ETB receptor expression [in arbitrary units (AU)] among YW (*n* = 15, closed circles) and PMW (*n* = 15, open circles) **(C)** Data from Kuczmarski et al. (2020).

There has been an increasing amount of evidence pointing to changes in ETB receptor function as a mechanism contributing to vascular dysfunction in women. Our lab has provided additional support demonstrating that the ETB receptor promotes vasodilation in young women however this effect is lost following menopause (Figure 2A) (Wenner et al., 2017). This switch in ETB receptor function in postmenopausal women suggests that the ETB receptor is a primary driver of ET-mediated vascular dysfunction in women (Wenner et al., 2017). Moreover, we have recently shown reduced ETB receptor expression on endothelial cells in postmenopausal compared to young premenopausal women (Figure 2B) (Kuczmarski et al., 2020). Additionally, we reported a positive relation between FMD and ETB receptor expression further suggesting a role for the ETB receptor in contributing to vascular function (Figure 2C). Indeed, these studies suggest there is attenuated ETB mediated dilation in postmenopausal women in part due to lower endothelial ETB receptor expression, and possibly an increased vascular smooth muscle cell ETB-mediated constriction.

The loss of estradiol (E2) during menopause could be a factor contributing to the changes in the ET system. Estradiol not only directly impacts ET-1, but also enhances NO and PGI_2_ (Davidge, 2001). To better understand the impact of sex hormones on the ET system we conducted a study examining ET receptor function during two stages of the menstrual cycle. In healthy young women, during the mid-luteal phase when estrogen and progesterone concentrations are elevated, ET receptor function favored a vasodilatory profile (Sebzda et al., 2018). Thus, both aging and loss of hormones could contribute to phenotypic and functional changes in the ET system in postmenopausal women. Additional work is needed to tease out the individual effects of estradiol and progesterone on ET receptor function.

The use of animal models, specifically ovariectomy (OVX), a form of surgical menopause that reduces levels of ovarian hormones in females, with E2 add-back, have been a useful tool to study the effects of E2 on the ET system. OVX leads to increased plasma and cellular ET-1 levels as well as increased ET-1 mediated constriction (Wang et al., 1997; Rodrigo et al., 2003; Tan et al., 2003). Increased VSMC constriction is mediated by increased ETA mediated tone and/or increased ETB receptor expression (Sudhir et al., 1997; David et al., 2001). E2 add-back following OVX attenuated ET-1 levels and ET-1 mediated constriction (Sudhir et al., 1997; Tan et al., 2003). Some of these actions appear to be through E2 receptors as treatment with the selective estrogen receptor modulator, raloxifene, reduced ET-1 expression compared to the OVX group (Demir et al., 2013). Additionally, both ET receptor antagonism and E2 independently or combined, have a vasoprotective effect in OVX rats following vascular injury (Kitada et al., 2014).

ET receptor expression is also impacted by E2. In an animal model using OVX and E2 add-back, the loss of ovarian hormones increased ET receptor expression, however this response was attenuated when E2 was restored (Gohar et al., 2016). ETA receptor mRNA expression, but not ETB receptor expression, was reduced after treatment with E2 in the aorta of OVX rabbits (Pedersen et al., 2009). In rat aortic VSMCs there was also a reduction in ETA receptor protein expression following E2 treatment; unfortunately, ETB receptor expression was not evaluated in this study (Ting-Huai et al., 2001). In contrast, cardiomyocytes from OVX spontaneously hypertensive rats had decreased ETB receptor mRNA expression following E2 treatment with no change in ETA receptor expression (Nuedling et al., 2003). Another study evaluating the impact of E2 following a myocardial infarction in female rats demonstrated no change in ETA receptor expression but attenuated ETB receptor expression in the left ventricle in the E2 treated group compared to placebo (Smith et al., 2000). Importantly, different tissues have different ratios of endothelin receptor expression, and the model used can also impact ET receptor expression. Gohar et al. examined ETA receptor and ETB receptor mRNA expression in OVX rats treated with E2 and demonstrated tissue specific changes in receptor expression. For example, in the lungs, E2 decreased ETA receptor expression but did not change ETB receptor expression, whereas in the liver, E2 did not change ETA receptor expression but increased ETB receptor expression (Gohar et al., 2016). It has also been shown that E2 attenuates ETA expression on VSMCs in culture (Ting-Huai et al., 2001). Thus, there appears to be tissue dependent actions of E2 on the ET system, such that loss of E2 can lead to dysregulation of the endothelin system and restoration of E2 seems to attenuate the ET system.

Investigation of the underlying mechanistic pathways to explain the role of E2 on the ET system are ongoing. In cell culture models, E2 treatment was able to attenuate ET-1 production in cells stimulated with numerous secretagogues including angiotensin-II, thrombin, TNF-α, and cyclic strain (Bilsel et al., 2000; Juan et al., 2004; Chao et al., 2005; Pearson et al., 2008; Morey et al., 2015). This could be due to E2 directly inhibiting ET-1 production through regulating gene transcription (Bilsel et al., 2000). E2 can act through both its ERα and ERβ receptor in HUVECs to attenuate ET-1 production (Evans et al., 2002). E2 can also modulate mast cell degranulation activity, thereby affecting the source of ET-1 modulating proteases (Maurer et al., 2004; Zierau et al., 2012). The addition of a pure estrogen receptor antagonist, ICI182780, attenuated the effects of E2 on ET-1 production demonstrating the presence of an estrogen receptor-mediated pathway (Akishita et al., 1998). Additionally, short term E2 exposure attenuated the vasoconstrictor effects of ET-1 in porcine coronary artery rings (Teoh et al., 2000). Collectively these studies suggest E2 can attenuate the production, secretion, and constrictor effects of ET-1.

More recently the G-protein estrogen receptor (GPER) has been shown to play a role in modulating the ET system by attenuating the ET-1 vasoconstrictor response in VSMC (Meyer et al., 2012) as well as increase NO production in endothelial cells (Fredette et al., 2018). GPER also can modulate ET-1 as well as ETA and ETB receptor expression in the kidney (Gohar et al., 2020). Indirect effects are mediated by E2 ability to increase NO, which can downregulate ET-1 production (Bilsel et al., 2000; Tan et al., 2007). When NO was inhibited, there was increased ET-1 production providing additional evidence of the regulatory effects of NO (Akishita et al., 1998). Receptor-independent mechanisms have been described using the E2 metabolites, 2-hydroxyestradiol and 2-methoxyestradiol, both of which have inhibitory effects on ET-1 release (Dubey et al., 2001). This finding could have important implications for postmenopausal women who can still produce E2 locally in various tissues. These different pathways suggest some redundancy in the ability of hormones and their metabolites to attenuate the ET system, and while favorable effects of E2 replacement therapy on plasma ET-1 have been reported in postmenopausal women (Best et al., 1998), the underlying mechanisms have yet to be studied in humans. This additional production pathway is an important consideration for women using menopausal hormone therapy (Bolton 2016; Naftolin et al., 2019).

### Men

**FIGURE 3:**
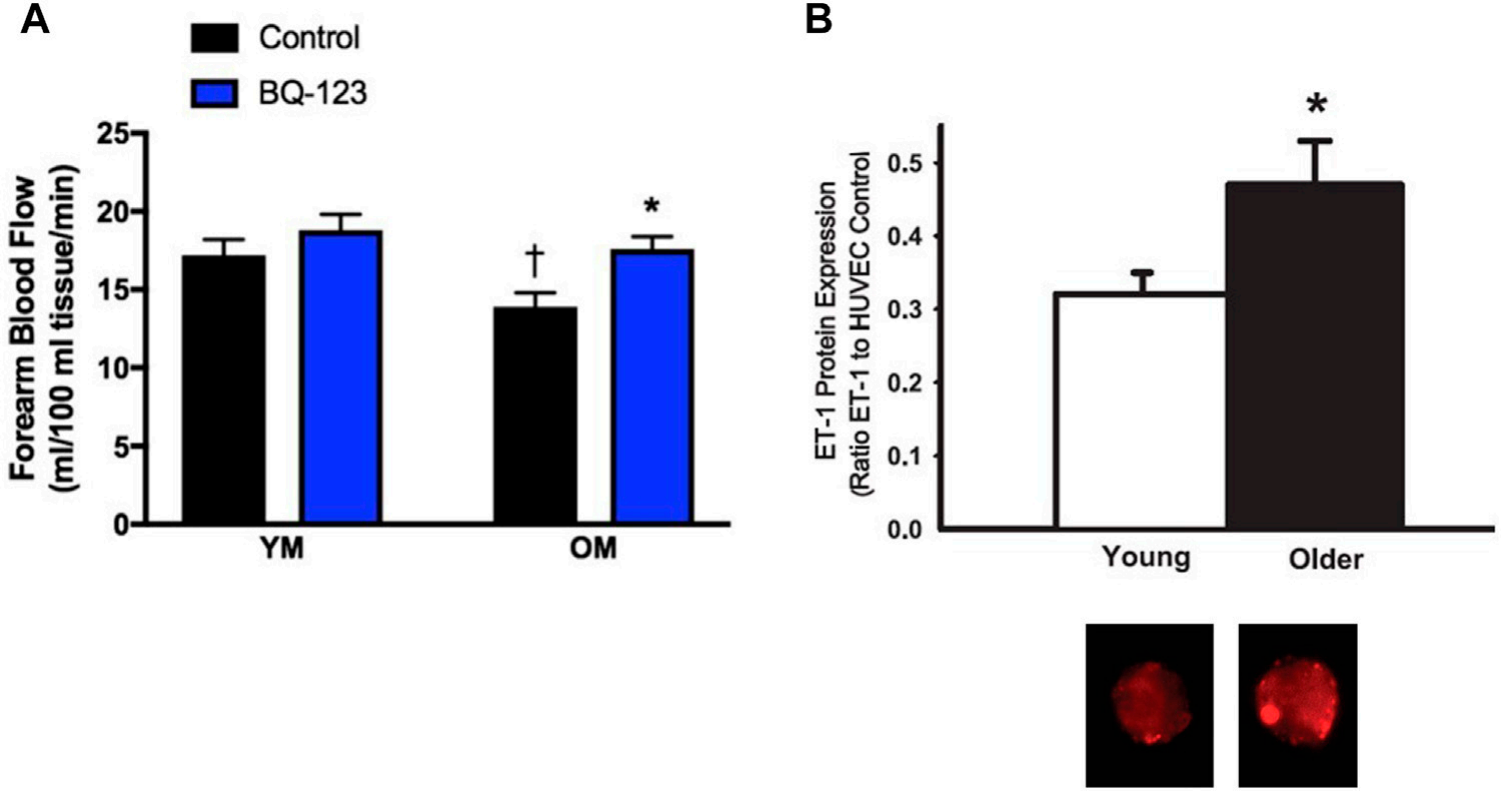
ETA-mediated vasoconstriction and increased cellular expression of ET-1 contributes to age-related declines in endothelial function in men. Forearm blood flow responses to acetylcholine were measured in young men (YM) and older men (OM) during saline conditions (control; black bars) or co-infused with the ETA receptor antagonist BQ-123 (blue bars). The age-associated decline in endothelial function was abolished with co-infusion of BQ-123 **(A)** Graph from Stanhewicz et al. (2018); data from Westby et al. (2011). Endothelial cellular expression of ET-1 (as a ratio of ET-1 to HUVEC control) collected in healthy young and old men. **(B, top)** and representative images of venous endothelial cell ET-1 expression obtained from brachial arteries in a young and older man **(B, bottom)** Data from Donato et al. (2009)).

The ET system also contributes to vascular dysfunction in men. In young men ETB and ETA receptors mediate vasoconstrictor tone (Haynes and Webb, 1994; Kellogg et al., 2001). In older men there is greater ETA mediated vasoconstrictor tone compared to younger men, as determined by brachial artery infusion of the ETA receptor antagonist, BQ-123 (Van Guilder et al., 2007). Furthermore, BQ-123 administration improved acetylcholine-mediated vasodilation in older men (Westby et al., 2011), suggesting that ETA-mediated vasoconstriction contributes to the age-related endothelial dysfunction in men (Figure 3A). Both plasma and endothelial cell ET-1 expression (Figure 3B) are increased in older men compared to younger men (Donato et al., 2009), and the vasoconstrictor response to exogenous ET-1 is attenuated in older men compared to younger men (Van Guilder et al., 2007). Thus, there appears to be elevated ET-1 bioavailability as well as increased ETA receptor activation leading to augmented ET-mediated vasoconstrictor tone in older men. These changes are likely due to aging and possibly declines in testosterone, both of which can modulate the ET system and vascular function.

To evaluate the impact of androgens on the vasculature, testosterone treatment has been used in animal models. In a study using aortas from male rabbits, testosterone was found to worsen endothelial function (Hutchison et al., 1997). This effect was also observed during treatment of porcine coronary arteries with testosterone, and further showed augmented vasoconstrictor responses to ET-1 (Teoh et al., 2000). Loss of testosterone through orchidectomy induced a significant increase in ET-1 and ETB receptor expression in the rat portal vein, which was reversed following testosterone replacement, however ETA receptor expression remained unchanged in the presence or absence of testosterone (Rossignoli et al., 2014).

Additional evidence of the impact of testosterone on the ET system is found in cell culture experiments. In human aortic endothelial cells, testosterone increased the number of cells secreting ET-1 and upregulated ET-1 mRNA within 3 h (Pearson et al., 2008). In porcine aortic and human coronary artery endothelial cells testosterone increased ET-1 release in both short term (5 h) and long-term incubation (24 h). Additionally, antagonizing the testosterone receptor using flutamide resulted in attenuated testosterone-mediated ET-1 release (Wilbert-Lampen et al., 2005). However, when haloperidol, a non-selective sigma-1 receptor antagonist was used in combination with testosterone treatment there was significantly less ET-1 produced. Thus, testosterone can act through direct and indirect pathways to increase ET-1 concentrations. The use of hormone therapies in men has been used for conditions such as hypogonadism where low testosterone can be detrimental, however, the use of testosterone therapy with natural aging is more controversial. A recent review of the impact of testosterone on vascular aging can be found here (Moreau et al., 2020).

## Pharmacological and Non-Pharmacological Therapies

Given that the ET system contributes to numerous vascular pathologies, its receptors are a target for pharmacological intervention. ET-receptor antagonists (ERA) have been studied in several pathologies; however, the studies were stopped early due to an increase in adverse effects of ERA treatment including increased fluid retention and liver toxicity, with the outcomes falling short of expectations (Ohkita et al., 2012; Dhaun and Webb 2019). Although at least eight small molecule ERA compounds have been developed, only three currently have FDA approval (bosentan, macitentan, and ambrisentan), and are only approved for pulmonary arterial hypertension (PAH). These ERAs are considered a first line therapy for PAH as they can improve symptoms and increase quality of life (Galiè et al., 2008), yet women experience greater benefits to ERAs compared to men (Gabler et al., 2012). Interestingly, the prevalence of PAH is ∼4:1 in women compared to men, and although the survival rate is higher in women, this drops after the age of 60 and is similar to that in men (Mair et al., 2014; Batton et al., 2018). These barriers with ERA efficacy have prompted the development of new, more selective ERAs.

New study designs have helped to increase efficacy by using an enrichment-responder design that identifies participants who demonstrate a response to the drug at low doses and who will potentially be less likely to have an adverse event. The SONAR trial was the first to use this approach in patients with type 2 diabetes and kidney disease, and demonstrated that in responders, Atrasentan, a selective low dose ERA targeting ETA receptors, reduced the risk of renal events (Heerspink et al., 2019). It was noted that there was high variability in the responses to Atrasentan, and in a sub-analysis of the SONAR trial there were sex differences in the pharmacokinetics of Atrasentan such that women had significantly higher area under the plasma concentration curve of plasma Atrasentan compared to men (Koomen et al., 2021). Additionally, there were racial differences observed in both Asian and Black patients compared with North American patients (Koomen et al., 2021). Indeed, given the sexual dimorphism of ET receptor subtype expression and function with aging, the impact of aging and sex on the efficacy of these selective ERA therapeutics should not be ignored.

Novel therapeutics targeting different parts of the ET system as well as novel ways to antagonize receptor function have shown promise in early studies. New methods using immunotherapy to create custom antibodies to ET receptors also show promise for the treatment of PAH. A specific antibody against the ETR-002 epitope of the ETA receptor was able to reduce right ventricle systolic pressure in PAH animals (Dai et al., 2019). Additionally, new molecular constructs, known as ET traps, have been developed which bind and sequester circulating ET-1 with the goal of reducing pathological levels to physiological levels (Jain, Coffey, et al., 2019), and have been shown to work in type 1 diabetics (Jain et al., 2019). These new approaches in drug development, disease selection, and trial design provide opportunities for creating targeted therapies focusing on the ET system.

### Exercise

Exercise training also has beneficial effects on health and longevity and can reduce CVD risk. Vascular endothelial function is reduced in middle-aged and older sedentary adults and aerobic exercise training can rescue these impairments. Given the distinct role of ET-1 on age-associated impairments in endothelial function in both men (Westby et al., 2011; Moreau et al., 2020) and women (Tan et al., 2003; Wenner et al., 2017), as well as populations with overt CVD risk factors such as hypertension (Nyberg et al., 2013) or overweight/obesity (Dow et al., 2017), the ability for exercise to reduce ET-1-mediated vascular dysfunction is an important area of study.

Clear evidence demonstrates aerobic exercise training improves ET system function in previously sedentary older men, and similarly, endothelial function is preserved in habitually active older men (DeSouza et al., 2000; Pierce et al., 2011). These findings can be explained, in part, due to the effects of exercise on the endothelin system. In young males, 8 weeks of aerobic exercise training decreased plasma ET-1 concentrations, that was reversed with 8 weeks of detraining (Maeda et al., 2001). Older men who have engaged in lifelong habitual physical activity also appear to have protection against elevations in plasma ET-1 with aging (Nyberg et al., 2013). Moreover, 12 weeks of aerobic exercise training in older sedentary men resulted in a reduction in ETA-mediated vasoconstrictor tone (Van Guilder et al., 2007). These data suggest a favorable effect of aerobic exercise in men to reverse age-related increases in ET-1 system activity and improve or preserve endothelial function with aging.

In women, the impact of aerobic exercise on endothelial function is equivocal, with data showing endothelial function is not consistently preserved (Pierce et al., 2011; Santos-Parker et al., 2017; Gliemann et al., 2020) in habitually active postmenopausal women, or improved after an aerobic training intervention (Pierce et al., 2011; Moreau et al., 2013; Gliemann et al., 2020). Although plasma ET-1 concentration is reduced in older postmenopausal women after aerobic exercise training (Maeda et al., 2003), greater reductions have been observed in premenopausal women (Nyberg et al., 2014). Moreover, the impact of aerobic exercise on ET-1-mediated vasoconstrictor tone in women is not yet known. Determining the sex- and age-specific differences in ET receptor subtype expression and function to modulate endothelial function after aerobic exercise training remains a rich area of research.

E2 is known to modulate the ET system as described above. Recent data indicate that E2 plays an essential role to “transduce” the benefits of exercise on the vasculature in postmenopausal women (Moreau et al., 2013). Therefore, it is likely that E2-deficient postmenopausal women demonstrate a general lack of vascular adaptations to aerobic exercise training compared to men due to loss of endogenous E2 production at menopause (Moreau et al., 2013). These vascular impairments are likely further exacerbated by a concomitant increase in ET-1 system activity and/or dysfunctional ET-1 receptors with age and/or menopause. Identifying windows of opportunity for preventative or early treatment, and optimizing exercise interventions that effectively protect against endothelial impairment, is critical for reducing CVD in women given the stark reduction in vasoprotective sex hormones and endothelial function along with a concomitant rise in prevalence of hypertension and CVD with menopause.

## Summary and Future Directions

**FIGURE 4:**
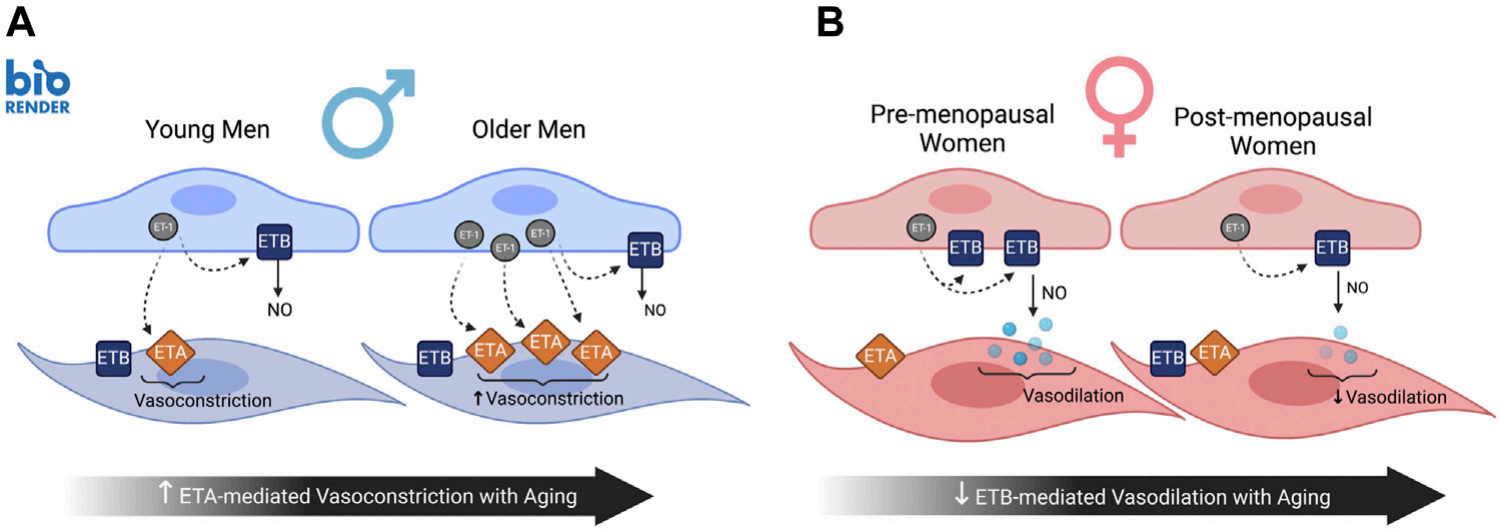
Sex differences in ET-1 signaling pathway with aging impacting endothelial function. In men, aging is associated with overexpression of cellular ET-1 and greater ETA receptor mediated vasoconstriction **(A)**, both of which contribute to declines in endothelial function. In contrast, the age-related decline in endothelial function in women is mediated in part by decreased endothelial ETB receptor expression and loss of ETB-mediated vasodilation **(B)**. Created with BioRender.com.

In summary, sex-specific differences in the ET-1 signaling pathway plays a large role in sex differences observed in endothelial function with aging (Figure 4). In women, aging is associated with a decreased endothelial ETB receptor expression (Kuczmarski et al., 2020) and loss of ETB-mediated vasodilation (Wenner et al., 2017) contributing to endothelial dysfunction (Wenner et al., 2017). These changes in ETB receptor expression and function are likely modulated by sex hormones based off work in cell culture and preclinical animal models, as well as emerging data in humans. In men, aging is associated with overexpression of cellular ET-1 and greater ETA receptor-mediated vasoconstriction, both of which contribute to declines in endothelial function (Figure 4). These sex-specific differences in ET receptor subtype expression and function can modulate ET-1 while also increasing vasoconstrictor tone and exacerbating its pressor effects. However, it remains unclear whether the age-related increases in plasma ET-1 are due to these aforementioned changes in expression of ET-1 or ETA/ETB receptors, or clearance by the ETB receptor, in humans. The local tissue concentrations of ET-1 and expression of ET-1 in VSMC as well as changes in ETA/ETB receptor expression on VSMC in aging men and women is an important area of future investigation. As vascular dysfunction becomes more apparent with aging, the ET system should not be overlooked as an important contributor to sexual dimorphism in the development and progression of age-associated CVD. Moreover, additional work is needed to understand if there is a shift due to aging and/or fluctuations in sex hormones in the different signaling pathways of ETA and ETB receptors.

Given the ET system is a critical player in regulating vascular function as well as numerous pathological conditions, the ability to regulate the ET system through lifestyle or pharmacological interventions are valuable therapeutic tools. Finding windows of opportunity for preventative or early treatment is critical to reducing CVD. More research is needed to develop new methods to manipulate production of ET-1 and receptor function as therapeutic targets, since currently approved drugs tend to have numerous side effects. Targeting specific ET receptors based on receptor subtype and tissue location could help to attenuate the ET system and help preserve vascular function without systemic side effects. Lifestyle changes are often first line treatments to prevent, delay, and reverse elevations in CVD risk. ET system activity may be an important mechanism underlying the sex-specific differences in adaptations to aerobic exercise and is an important question that warrants future study. This is critical given that less than 1 in 4 adults report sufficient activity to meet the relevant national physical activity guidelines for both aerobic and muscle strengthening exercise (Gliemann et al., 2020). Finally, understanding the effects of hormone therapy in men and women and the timing of initiating treatment on the ET system may provide valuable information for preventing or delaying the age-associated declines in endothelial function and increased prevalence of CVD.
